# Prevalence of chronic kidney disease in diabetic adult out-patients in Tanzania

**DOI:** 10.1186/s12882-016-0276-9

**Published:** 2016-07-08

**Authors:** Bonaventura C. T. Mpondo, Eric Neilson, Alex Ernest

**Affiliations:** Department of Internal Medicine, School of Medicine and Dentistry, College of Health and Allied Sciences, The University of Dodoma, Dodoma, Tanzania; Peace Corps/SEED Global Health Service Partnership, Massachusetts General Hospital Center for Global Health, Boston, MA USA; Department of Surgery and Maternal Health, School of Medicine and Dentistry, College of Health and Allied Sciences, The University of Dodoma, Dodoma, Tanzania

**Keywords:** Diabetes mellitus, Schistosomiasis, Microalbuminuria, Proteinuria, Chronic kidney disease

## Abstract

Despite the increasing number of patients with Diabetes Mellitus in sub-Saharan Africa, the magnitude of chronic kidney disease among diabetics has not been well established. A study done by Janmohamed et al. found chronic kidney disease in 83.7 % of diabetics which is relatively higher than the prevalence reported elsewhere. However this study was conducted in schistosoma endemic area along the shores of Lake Victoria. Schistosomiasis has been reported to cause a range of renal diseases. Interpretation of these findings should therefore take into account the possibility of schistosomiasis as a possible confounder.

## Correspondence

We have read the article by Janmohamed et al. [1] published in your journal with much pleasure and interest. The authors enrolled 369 adult diabetic out-patients and screened them for renal dysfunction by measuring proteinuria, microalbuminuria and serum creatinine. In this study, the authors found that 83.7 % of the study participants had chronic kidney disease (CKD) defined as an estimated glomerular filtration rate (eGFR) ≤ 60 ml/min/1.73 m^2^ or having evidence of kidney damage (microalbuminuria or overt proteinuria). The prevalence of albuminuria was found to be 80.0 % in this cohort. Out of the patients with albuminuria, 45.8 % had microalbuminuria alone and 34.1 % of the participants had overt proteinuria. This study was conducted in a tertiary level hospital located in Mwanza, Tanzania, in the Lake Victoria zone.

The rates of overt proteinuria found in this study were higher than those observed in other similar studies in sub-Saharan Africa [2–4]. Another study done in a different tertiary hospital in Dar es Salaam Tanzania found the overall prevalence of microalbuminuria to be 10.7 % and macroalbuminuria to be 4.9 %. In this study, patients with type 1 diabetes were found to have microalbuminuria at a rate of 12 % and macroalbuminuria at a rate of 1 %. Patients with type 2 diabetes were found to have microalbuminuria at 9.8 % and macroalbuminuria at 7.2 % [5]. The rates are significantly lower when compared to the study by Janmohamed et al.

The study by Janmohamed et al. was conducted in Mwanza, Tanzania, located along the shores of Lake Victoria. Previous studies have demonstrated a high prevalence of Schistosomiasis infection in this region [6–8]. The role of Schistosomiasis in causing renal disease has been well established. Existing reports show that Schistosomiasis is associated with a range of renal diseases from cystitis to immune mediated glomerulopathy [9–12].

Other studies assessing renal dysfunction in this region have shown relatively higher rates of renal dysfunction compared to studies elsewhere [13]. This study investigating HIV-infected patients initiating ART also did not screen for Schistosomiasis. In children, a study assessing the prevalence of renal dysfunction among HIV-infected and uninfected patients found that Schistosomiasis was a strong predictor of renal insufficiency in both HIV-infected and HIV-uninfected individuals [14]. These findings demonstrate that, in Schistosomiasis endemic areas, the high prevalence of renal insufficiency can be, at least partially, due to the parasitic infection.

The authors failed to mention the possible contribution of Schistosomiasis as a likely confounder in the observed high prevalence of renal insufficiency. In interpreting these results, it is important to take into consideration the high prevalence of this disease in the study setting and its possible role in causing renal diseases. The failure to screen for Schistosomiasis should have been mentioned as a one of the limitations of their study.

## Abbreviations

ART, antiretroviral therapy; CKD, chronic kidney disease; eGFR, estimated glomerular filtration rate; HIV, human immunodeficiency virus
